# Utilizing clinical pathways and web-based conferences to improve quality of care in a large integrated network using breast cancer radiation therapy as the model

**DOI:** 10.1186/s13014-018-0995-0

**Published:** 2018-03-16

**Authors:** Katherine S. Chen, Scott M. Glaser, Allison E. Garda, John A. Vargo, M. Saiful Huq, Dwight E. Heron, Sushil Beriwal

**Affiliations:** 1 University of Pittsburgh School of Medicine, Pittsburgh, PA USA; 2 Department of Radiation Oncology, UPMC Hillman Cancer Center, Pittsburgh, PA USA; 3 Department of Radiation Oncology, Mayo Clinic, Rochester, MN USA; 4 Department of Radiation Oncology, Magee-Womens Hospital of UPMC, Pittsburgh, PA USA

**Keywords:** Clinical pathway, Hypofractionation, Dose homogeneity, Web-based conference, Quality improvement, Dosimetric compliance, Education

## Abstract

**Background:**

Clinical pathways outline criteria for dose homogeneity and critical organ dosimetry. Based upon an internal audit showing suboptimal compliance with dosimetric parameters in whole breast irradiation (WBI), we conducted a mandatory web-based teaching conference for the network. This study reports the impact of this initiative on subsequent treatment plans.

**Methods:**

Radiation treatment plans were collected for the 10 most recent patients receiving WBI at 16 institutions within the UPMC Hillman Cancer Center network. Subsequently, a web-based conference was conducted to educate staff physicians, physicists, and dosimetrists with goals for dose homogeneity and critical organ dosimetry. Six months post-conference, another 10 plans were collected from each site and compared to pre-conference plans for deviations from dosimetric criteria.

**Results:**

Dose homogeneity significantly improved after the conference with breast V_105%_ decreasing from 15.6% to 11.2% (*p* = 0.004) and breast V_110%_ decreasing from 1.3% to 0.04% (*p* = 0.008). A higher percentage of cases were compliant with dosimetric criteria, with breast V_105%_ > 20% decreasing from 22.5% to 7.5% of cases (*p* = 0.0002) and breast V_110%_ > 0% decreasing from 13.8% to 4.4% of cases (*p* = 0.003).

**Conclusions:**

Implementation of a web-based teaching conference helped improve adherence to clinical pathway dosimetric guidelines for WBI. In radiation oncology networks, this may be an effective model to ensure quality in routine practice and can be extrapolated to other disease sites.

## Background

The technique of adjuvant whole breast irradiation (WBI) has improved significantly in the last decade. Modalities such as three-dimensional conformal radiation therapy (3D-CRT) and intensity modulated radiation therapy (IMRT) allow for assessment of dose distribution for the entire target volume, as opposed to only the central axis. Improved dose homogeneity in breast irradiation has been shown to significantly decrease the risk of moist desquamation and improve long-term cosmesis [1–6]. There is also strong evidence that increased mean heart dose linearly increases risk for major coronary events, which is particularly important for left-sided tumors [7, 8]. Given these outcomes, dose homogeneity and critical organ dosimetry have become important targets to achieve in treatment planning.

The UPMC Hillman Cancer Center is a National Cancer Institute-designated Comprehensive Cancer Center comprised of 4 central academic centers and 16 regional community centers. Within such a large network, variations in practice patterns are almost unavoidable. In order to help standardize clinical practices, the UPMC Hillman Cancer Center implemented clinical pathways for breast cancer in 2003. Clinical pathways apply evidence-based treatment-specific guidelines in the form of a decision support tool, and for breast cancer, they specify criteria for dose homogeneity and critical organ dosing in adjuvant radiation treatment. Based upon an internal audit showing suboptimal compliance with dosimetric parameters, we conducted a mandatory web-based teaching conference for the network. In this study, we report the impact of this teaching initiative on multi-center compliance with dosimetric guidelines across a large, integrated radiation oncology network.

## Methods

An internal audit was performed at 16 regional facilities in Western Pennsylvania within the UPMC Hillman Cancer Center network, including 3 academic sites and 13 community sites. The 10 most recent treatment plans from each of the facilities (*n* = 160) were obtained from integrated sites through the ARiA record-and-verify database (Varian Medical Systems, Palo Alto, CA) using International Classification of Disease, ninth revision, codes for breast cancer (174.0–174.9) and DCIS (233.0) between June 2015 and June 2016.

Clinical pathways (Via Oncology, Pittsburgh, PA) have been used in practice in the UPMC Hillman Cancer Center network since 2003, with implementation and continued review of guidelines as detailed in prior publications [9]. Pathway criteria for dose homogeneity and critical organ dosimetry were defined based on published data and critical evaluation of breast plans in our network [1–6, 10]. The criteria are stated as follows:

1. V_105%_ ≤ 10–15%, where V_105%_ is the breast volume receiving 105% of the prescription dose. Larger breasts can accept V_105%_ up to 15–20%, where 20% is the cutoff for meeting compliance.
2. V_110%_ = 0%, where V_110%_ is the breast volume receiving 110% of the prescription dose.
3. Mean heart dose < 3 Gy. The heart was contoured as an organ at risk for both left and right breast irradiation.

Following analysis of the baseline 160 plans, a mandatory web-based teaching conference for radiation oncologists, physicists, and dosimetrists was conducted across all 16 in-network oncology centers. Attendance was recorded, and a second session was conducted the following week for those who could not attend the first meeting. Conference content included a review of established contouring guidelines, dosimetric constraints, and network-wide preferences for treatment planning. Three-dimensional field-in-field (3D-FIF) radiation therapy without wedges was the recommended technique, with the rationale of reducing hot spots and minimizing scatter to the contralateral breast. If 3D-FIF treatment plans did not meet criteria for previously outlined coverage and dose homogeneity constraints, then tangential beam IMRT with inverse planning was recommended. Afterwards, a teaching document referencing the contouring and dosimetric criteria found in clinical pathways was distributed to all sites. Conference attendees were not notified that a follow-up audit would be conducted, nor were there any changes in incentives or penalties associated with pathway deviations.

The impact of web-based teaching was assessed 6 months after the conference. The 10 most recent breast plans from each of the 16 oncology sites were collected and analyzed for dose homogeneity and critical organ dosimetry. Pre-conference and post-conference plans (*n* = 320) were then compared as described below.

IBM SPSS version 22 (IBM cooperation, Armonk, NY) was used for data analysis. Variations in breast plans were analyzed based on the percentage of cases with breast homogeneity deviations for breast V_105%_ and V_110%_ or mean heart dose deviations. Subset analyses were performed for left versus right breast heart dose and for changes in dose homogeneity stratified by breast volume using the Mann-Whitney U test. Technique specification (3D-FIF vs. IMRT), energy utilization (6 MV vs. 6 MV + high energy), and factors predictive of dosimetric deviations were analyzed between pre-conference and post-conference using Chi-square analysis.

## Results

Baseline patient characteristics can be found in Table 1. There were no significant differences between the pre- and post-conference populations in terms of disease laterality, median breast CTV, and hypofractionation adoption.

**Table 1:** Baseline characteristics. (CTV, clinical target volume)

	Pre-conference	Post-conference	*p*-value
Breast Laterality			
Left	54.5%	48.1%	0.263
Right	45.5%	51.9%	
Breast CTV median (cc)	900.53	925.70	0.673
Hypofractionation			
Yes	72.5%	80%	0.115
No	27.5%	20%	

**Fig. 1:**
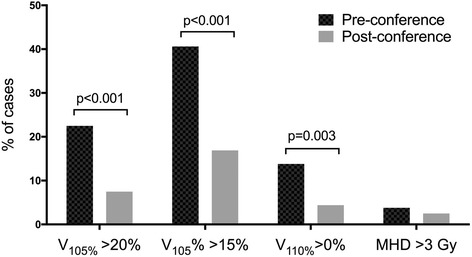
Changes in percentage of cases with dosimetric criteria deviations after the web-based teaching conference

V_105%_ and V_110%_ were used to assess changes in dose homogeneity pre- and post-conference, representing the breast volumes receiving 105% and 110% of the prescribed dose, respectively. After the conference, mean V_105%_ decreased from 15.6% to 11.2% (*p* = 0.004), and V_110%_ decreased from 1.3% to 0.04% (*p* = 0.008). There were also significant improvements in adherence to the thresholds recommended in the clinical pathways. Breast V_105%_ > 20% decreased from 22.5% of cases to 7.5% of cases (*p* = 0.0002), breast V_105%_ > 15% decreased from 40.6% of cases to 16.9% of cases (*p* < 0.0005), and breast V_110%_ > 0% from 13.8% of cases to 4.4% of cases (*p* = 0.003) (Fig. 1). Additionally, Table 2 details the changes in dose homogeneity after the conference as stratified by breast size. In terms of critical organ dosimetry, mean heart dose > 3 Gy was seen in 3.8% of cases pre-conference and 2.5% of cases (*p* = 0.52). In cases of left breast irradiation, median mean heart dose was 1.3 Gy pre-conference and 1.1 Gy post-conference. The mean heart dose was even lower in right breast irradiation, at a median of 0.40 Gy both pre- and post-conference. Factors predictive of breast dose homogeneity deviations (V105% > 20% and V110% > 0%) were pre-conference status (OR = 3.34, (1.77–6.29), *p* = 0.0002) and increased breast size (OR = 1.06 per 100 cc, (1.01–1.11), *p* = 0.016).

**Table 2:** Changes in V_105%_ after the conference as stratified by breast size. (CTV, clinical target volume; IQR, interquartile range)

	V_105%_ (%) (median, IQR)		
Breast CTV (cc)	Pre-conference	Post-conference	*p*-value
< 750 (*n* = 134)	10.0 (0.5–19.0)	9.7 (5.1–13.6)	0.595
750–1499 (*n* = 151)	13.9 (7.4–18.8)	10.4 (8.0–13.3)	0.035
≥1500 (*n* = 35)	15.0 (8.2–23.6)	12.5 (6.0–14.5)	0.228

The use of 3D-FIF decreased significantly from 128 cases (80%) pre-conference to 101 cases (63%) post-conference (*p* = 0.001). There was a corresponding increase in tangential beam IMRT from 32 cases (20%) pre-conference to 59 cases (37%) post-conference (OR = 2.34 (1.41–3.86), p = 0.001) in order to achieve improved dose homogeneity. Likewise, mixed energy (6 MV + high-energy) utilization increased from 83 cases (51.9%) pre-conference to 105 cases (65.6%) post-conference (OR = 1.77 (1.13–2.78), *p* = 0.013).

## Discussion

In radiation oncology, major variations in quality often occur during treatment planning and delivery, and these deviations are hard to capture. Despite the specification of parameters for dose homogeneity and critical organ dosimetry in our clinical pathways, an internal audit showed that baseline adherence to dose homogeneity was suboptimal, with 22.5% of treatment plans having breast V_105%_ > 20% and 13.8% of plans having V_110%_ > 0%. We hypothesize several potential barriers that may have affected compliance with clinical pathway recommendations. For physicians, physicists, and dosimetrists who initially had trained with 2D-RT for breast cancer, there may have been a general unfamiliarity with the importance of dose homogeneity, as much of the data to support dose homogeneity has come out in the last 10 years [1–6, 11]. Indeed, dose homogeneity guidelines themselves are not well defined in the literature. The only recommendation made in ASTRO guidelines for WBI is to limit maximum breast dose to less than 107%, on the basis of randomized trials involving standard 2D-RT planning [12].

We designed the network-wide educational intervention to address these possible uncertainties, and we can identify several key factors that led to its success. The web-based platform maximized outreach to all sites in the network, and we mandated the conference for not only physicians, but also physicists and dosimetrists. This helped ensure that all involved in the radiation planning process would be equally aware of expectations, and detailed technical guidance was provided on how best to achieve the dosimetric goals that were set. As a result, a significant improvement was seen in dose homogeneity following this education, with adherence to breast V_105%_ < 20% and V_110%_ = 0% in 92.5% and 95.6% of plans, respectively.

There are limitations and areas for future improvement for this intervention. This study was unable to account for operator-dependent variability, either between facilities or individual providers, but there were substantial network-wide improvements regardless. Although staff were not told plans would be re-analyzed, we are unable to account for post-conference changes that may have occurred secondary to a perceived observer effect. Similar interventions in the future may benefit from post-conference surveys assessing what factors or attitudes changed to produce this improvement in quality. To effectively stress the need for improvement, future educational strategies may also benefit from quantitative visualizations of group performance metrics. Finally, this study does not address the durability of the changes made post-conference, but long-term outcomes may be the subject of future studies.

## Conclusions

The web-based teaching conference is an effective model to improve adherence to clinical pathway guidelines in a large radiation oncology network. The goal of implementing these models is to try and achieve the same level of adherence as that seen in clinical trials, and analyses of phase III clinical trials have shown that major RT deviations had adverse impacts on toxicities, tumor control, and even survival outcomes [13–16]. Post-conference, we saw a significant improvement in adherence to dose homogeneity criteria in breast plans, and we are working to extend this approach to other disease sites. On the foundation of existing clinical pathways, the web-based conference encouraged practitioners to minimize deviations from dosimetric guidelines and improve quality of care significantly, helping ensure that outcomes seen in clinical studies translate to everyday practice.

Data were presented as an oral presentation at the 2016 American Society for Radiation Oncology (ASTRO) annual meeting in San Antonio, TX.

## Abbreviations

3D-CRT: Three-dimensional conformal radiation therapy
3D-FIF: Three-dimensional field-in-field
IMRT: Intensity modulated radiation therapy
V_105%_: Breast volume receiving 105% of the prescription dose
V_110%_: Breast volume receiving 110% of the prescription dose
WBI: Whole breast irradiation
